# Efficacy of Baduanjin for treatment of fatigue: A systematic review and meta-analysis of randomized controlled trials

**DOI:** 10.1097/MD.0000000000034707

**Published:** 2023-08-11

**Authors:** Haoyu Liu, Siling Liu, Lu Xiong, Bingquan Luo

**Affiliations:** a Capital University of Physical Education and Sports, Haidian, Beijing, China; b School of Sport and Art, Shenzhen Technology University, Shenzhen, Guangdong province, China; c Jiangxi Institute of Applied Science and Technology, Nanchang, Jiangxi province, China.

**Keywords:** Baduanjin, fatigue, meta-analysis, randomized controlled trials, systematic review

## Abstract

Numerous studies have suggested that Baduanjin, a traditional Chinese exercise, can alleviate fatigue symptoms in patients with various illnesses. The aim of this review was to evaluate the efficacy of Baduanjin in reducing fatigue symptoms.

**Methods:**

A comprehensive literature search was conducted using several databases, including PubMed, Web of Science, Embase, Medline, China Biology Medicine disc, China National Knowledge Infrastructure, and Wanfang, from inception to June 2023. Relevant studies reporting on the effects of Baduanjin on fatigue symptoms were included. A random-effects meta-analysis model with standardized mean differences was used to estimate the treatment effects. Moderator analyses were conducted using continuous variables and meta-regression. This review was registered in the International Prospective Register of Systematic Reviews (identifier CRD42023411532). Grading of recommendations, assessment, development and evaluations framework was used to assess the certainty of evidence.

**Results:**

Ten randomized controlled trials with patients diagnosed with 9 different diseases were included in the meta-analysis. The Baduanjin intervention groups showed significant improvements in total fatigue intensity (standard mean difference = −0.49, 95% confidence interval = −0.69 to −0.30, *P* = .000; *I^2^* = 56%, *P =* .009). The statistically significant differences in the subgroup analyses, including intervention durations, age of participants, fatigue types, and practice location, remained unchanged. Meta-regression showed that practice place might have significant effect on the results. The certainty of the evidence was moderate for participants 55-year younger or in hospital training. However, fatigue, different groups, participants 55-year or older, training at home, and different fatigue types had lower evidence certainty.

**Conclusion:**

Baduanjin can effectively alleviate fatigue symptoms with relatively flexible requirements. However, studies investigating the same disease types and including non-Chinese populations are scarce. Therefore, further studies with long-term interventions, larger sample sizes, and well-designed methodologies are warranted.

## 1. Introduction

Fatigue is a complex symptom that encompasses a range of complaints including lethargy, malaise, lassitude and exhaustion, resulting in significant impairment of the ability to function at the usual capacity.^[1]^ It can manifest in various forms, such as physical exhaustion, mental fatigue, or emotional burnout, and can have a profound impact on the individual overall well-being and quality of life.^[2]^ Furthermore, human fatigue is associated with a range of symptoms, including low mood, reduced vitality, boredom, and sleep disturbances. In severe cases, individuals may also experience muscle pain, weakness, and weight loss.^[3,4]^ Disease-related fatigue, which can be caused by the illness or its treatment, is characterized by a persistent feeling of exhaustion. Among various types of disease-related fatigue, cancer-related fatigue is the most prevalent and can significantly affect patients’ quality of life.^[5,6]^

To alleviate fatigue, patients often receive a combination of alternative and complementary treatments, such as scientific sports training, nutritional supplements, and traditional Chinese medicine.^[7,8]^ There are two main categories of methods for improving fatigue in patients: pharmacological and nonpharmacological interventions. However, the effectiveness of some drugs used to treat fatigue remains unclear and requires further investigation.^[9]^ In addition, certain medications may have adverse effects that could complicate the patient condition.^[10,11]^ As an alternative to pharmacological interventions, aerobic exercise has been recommended for treating fatigue.^[12,13]^ Physical exercises can not only relieve fatigue raised by cancerous diseases, physical exercises also have potentials suppressing hepatocellular carcinoma progression by alleviating cancer stemness.^[14–17]^

According to traditional Chinese medicine, Baduanjin qigong has multiple health benefits, including promoting the smooth flow of meridians, reinforcing Qi, promoting blood circulation, regulating visceral function, strengthening the body immunity, and expelling harmful toxins.^[18,19]^ These effects can improve sleep, mood regulation, and management of blood lipid levels. Moreover, regular practice of Baduanjin qigong can help individuals to maintain their physical fitness and overall well-being.^[20,21]^ The Baduanjin is a set of aerobic exercises that are believed to improve overall health and promote a smooth flow of energy throughout the body. Baduanjin is comprised of 8 distinct exercises, each focusing on different physical areas and meridians,^[22]^ and emphasizes the cultivation of both physical and psychological well-being through balanced maintenance of body and mind.^[23]^ While Baduanjin, yoga, tai chi, and breathing training all involve elements of mindfulness and meditation, they differ in terms of specific movements, postures, and focuses. Yoga emphasizes physical postures, breathing exercises, and meditation to promote flexibility, strength, balance, and mental well-being.^[24,25]^ Tai chi involves slow and controlled movements, deep breathing, and mental focus to promote balance, flexibility, and relaxation.^[26,27]^ Breathing training specifically focuses on improving breathing techniques to enhance respiratory function and promote relaxation.^[24]^ Wuqinxi emphasizes the inter-coordination of body movement, breathing, and meditation,^[28]^ while Baduanjin emphasizes the feeling of movement and breathing, and the most prominent feature is that it is composed of 8 simple movements, which is very suitable for ordinary people to practice.

The preceding meta-analytic studies^[29–32]^ frequently combined Baduanjin with other forms of qigong or failed to perform detailed subgroup analyses tailored to Baduanjin. Consequently, the unique movement and breathing features of Baduanjin qigong were often overlooked. Furthermore, these studies have disproportionately emphasized the impact of Baduanjin on cancer-related fatigue,^[33,34]^ disregarding its effectiveness in other populations. Consequently, the results generated do not furnish comprehensive guidelines for ameliorating fatigue in different cohorts. While certain studies have shown that Baduanjin exercises exhibit a salutary impact in mitigating disease-associated fatigue, inconsistent findings have been reported in other studies.^[35,36]^ Furthermore, there is no consensus on the effect of Baduanjin on fatigue under different duration, age of subjects, practice location, and fatigue type. In this study, we aimed to conduct a quantitative analysis of randomized controlled trials (RCTs) to evaluate the efficacy of Baduanjin for fatigue treatment in adults. In addition, this study intended to determine the optimal intervention strategies, including sex, age, intervention duration, fatigue type, or practice location, and furnish recommendations for clinical implementation.

## 2. Methods

Our meta-analysis was conducted according to the Preferred Reporting Items for Systematic Reviews and Meta-Analyses guideline.^[37]^ This systematic review and meta-analysis were registered in International Prospective Register of Systematic Reviews (registration number: CRD42023411532).

### 2.1. Search methods

A comprehensive literature search was conducted using 7 databases, including PubMed, Web of Science, Embase, Medline, China Biology Medicine disc, China National Knowledge Infrastructure, and Wanfang, from inception to May 2023. We conducted searches using relevant keywords, including baduanjin, buduanjin, 8-section brocades, 8-section exercises, 8-treasured exercises, fatigue, and random*. The inclusion criteria were as follows: RCT design; participants aged ≥18 years with or without diseases; comparison of Baduanjin with any type of control; assessment of fatigue as the outcome; and publication in English or Chinese language. Discrepancies were discussed and resolved between 2 reviewers, HL and SL, and any unresolved issues were consulted with a third reviewer, BL. To ensure a comprehensive literature search in Chinese databases, including China National Knowledge Infrastructure, Wanfang, and CAM SinoMed, we used relevant Chinese terms such as “八段锦 (Baduanjin)” for a specific exercise routine, “疲劳 (fatigue)” to indicate the specific outcome of interest, and “随机 (random*)” to cover studies that use randomization. In addition, we manually searched the reference lists of the included studies and relevant reviews to identify additional studies that met our inclusion criteria. Detailed search tactics are shown in Supplementary Appendix 1, http://links.lww.com/MD/J459.

### 2.2. Inclusion and exclusion criteria

1. Types of studies: This research only included RCTs published in English or Chinese. To be considered as an RCT, the study had to allocate participants randomly to either the experimental or control group. Repeated publications, review articles, study protocols, survey studies, meta-analyses, dissertations, and narrative studies were excluded.
2. Types of participants: Participants of 18-year-old or older were included.
3. Types of intervention: Studies included in this review used Baduanjin exercises in the intervention group and no specific interventions in the control group. Studies that combined Baduanjin exercises with other forms of intervention or used alternative forms of intervention were excluded from the analysis.
4. Types of outcome measures: The outcome measures were scales that measured the effects on fatigue.

### 2.3. Study screening and data extraction

Two reviewers (HL and SL) evaluated the studies based on titles, abstracts, and full texts. Any disagreements were resolved through discussion and consensus between HL and SL. If inclusion remained unclear, a third reviewer, BL, was consulted. Two reviewers extracted and summarized relevant data, such as leading authors’ names, publication year, country, language, initial sample size, dropout rate, study population, cause for fatigue, fatigue type, participant characteristics (such as disease condition, mean age, and sex ratio), intervention dosage, outcome measure tools, and practice location.

### 2.4. Assessment of quality of literature

To evaluate the quality of each included study, 2 independent researchers utilized the revised Cochrane risk-of-bias tool for RCTs.^[38]^ The tool evaluated several characteristics, including random sequence generation (selection bias), allocation concealment (selection bias), blinding of participants and personnel (performance bias), incomplete outcome data (attrition bias), selective reporting (reporting bias), and other biases. The resulting data were graphed and analyzed using Review Manager 5.3. Each assessment was conducted by 2 independent reviewers (HL and LX), and any differences were resolved by consultation (HL and LX) or the assistance of a third author (BL).

### 2.5. Data analysis

The analysis in this study was conducted using Review Manager 5.3, which employed random effects modeling (weighted by the inverse of the variance) to synthesize traditional pairwise efficacy data in populations. The efficacy of continuous data of fatigue, which were measured using different tools, was assessed using the standard mean difference (SMD) and 95% confidence interval (CI). Statistical significance was set at *P* < .05, unless stated otherwise.

We used *I*^2^ statistic and *P* value to assess heterogeneity and random effects model to analysis. Sensitivity analyses were performed to examine the stability of the pooled outcomes, and meta-regression analyses were conducted for exploring potential sources of heterogeneity. Subgroup analyses based on intervention durations, age of participants, fatigue types, and practice location were also conducted in a predesigned manner.

To further analyze the influence of heterogeneity on the meta-analyses’ conclusions, meta-regression and subgroup analyses were conducted. These analyses explored the primary outcome data based on duration length, single intervention time, total time, number of interventions, proportion of females, age, practice place, fatigue type.

A qualitative assessment of potential publication bias was conducted using funnel plots, which were visually inspected for asymmetry. Sensitivity analysis were performed using Hedges’g statistic. Statistical assessment of publication bias were performed using the Egger weighted regression method. All these statistical analyses were performed using STATA 11.0 (Stata-Corp).

### 2.6. Certainty of evidence

For outcomes included in meta-analyses, certainty of the evidence was assessed using the grading of recommendations assessment, development and evaluation (GRADE) framework^[39]^ by 2 independent reviewers (HL and LX). GRADEpro Guideline Development Tool software (McMaster University and Evidence Prime) and its guidance on thresholds for each domain were used to conduct the assessment. There were no disagreements, and an arbitrator was not used.

## 3. Results

### 3.1. Literature search

**Figure 1.**
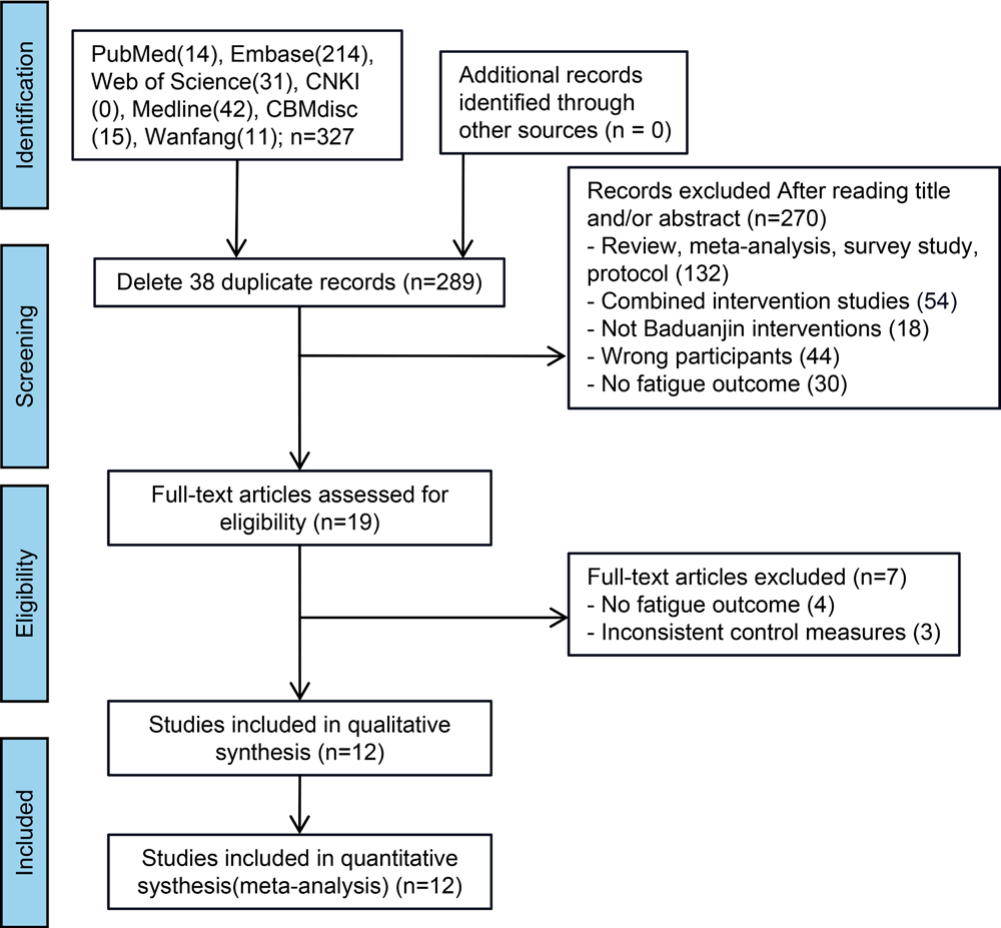
Screening process for trials included in the meta-analysis.

Overall, we found 327 related articles through online databases; 38 repetitive articles were excluded. After screening titles and abstracts, we further excluded 132 non-RCT studies, 54 studies that utilized an combined interventions, and 30 studies that did not have fatigue outcome measures. We acquired the full text of 19 articles and eliminated 7 of them for the following reasons: 4 for no fatigue outcome, and 3 for inconsistent control measures (Fig. 1). Finally, 12 qualifying articles published between 2014 and 2023, comprising 1051 participants, were analyzed in this meta-analysis.

### 3.2. Characteristics of publications

All 12 studies and participants included were from China.^[35,36,40–47]^ Participants were all adults aged 18 to >70 years. Of 12, only 1 study had exclusively female participants.^[46]^ All the studies did not have an equal male-to-female ratio. A total of 11 fatigue test scales were used in the included studies, including the European Organization for Research and Treatment of Cancer Quality-of-Life Questionnaire Core 30, Brief Fatigue Inventory, Multidimensional Fatigue Inventory-20, Multidimensional Fatigue Symptom Inventory-Short Form, Chalder Fatigue Scale, modified Piper Fatigue Scale, Profile of Mood States, Fatigue Scale-14, and Fatigue severity scale. Two studies had an intervention completion rate of <80%.^[43,48]^ Therefore, the Baduanjin intervention has high feasibility. The details of intervention information of Baduanjin and outcome measure scales are displayed in Table 1.

**Table 1:** Main characteristics of studies.

Study	Study population	Cause for fatigue	Fatigue type	N (Total, female/male)	Drop-out rate (Experimental group/control group)	Practice location (home/hospital)	Age (Qigong group/control group, Mean ± SD, yr)	Intervention content	Control group	Duration, sessions with supervision per wk, time per session	Measurement tools of fatigue
Jingwen et al 2022^[46]^	Patients	Breast cancer , Aromatase inhibitors therapy	Pathological fatigue	68 (68/0)	8.33%/2.78%	Hospital	53.12 ± 7.02/54.63 ± 8.44	Baduanjin	Usual care	12 wk, 2, 90 min	EORTC QLQ-C30
Lu et al 2019^[40]^	Patients	Colorectal cancer and chemotherapy	Pathological fatigue	87 (31/56)	4.44%/2.22%	Hospital	55.60 ± 11.23/54.63 ± 11.88	Baduanjin	Usual care	24 wk, ≥5, 20–40 min	BFI
Liying et al 2023^[35]^	Patients	Nasopharyngeal carcinoma , radiotherapy combined with chemotherapy	Pathological fatigue	88 (21/67)	18.2%/11.4%	Hospital	45.55 ± 8.99/47.07 ± 9.43	Baduanjin	Usual care	12 wk, 5,40 min	MFI-20
Xiaolin et al 2021^[41]^	Patients	Breast cancer and chemotherapy	Pathological fatigue	70 (70/0)	8.6%/0%	Hospital	35 ± 4.25/35 ± 3	Baduanjin	Usual care	12 wk, 5, 30 min	MFSI-SF
Chan et al 2014^[42]^	Patients	Chronic fatigue syndrome-Like illness	Pathological fatigue	150 (75/75)	0%/0%	Home	39.1 ± 7.8/38.9 ± 8.1	Baduanjin	Usual lifestyle	9 wk, 16 sessions in total, 90 min/session	ChFs
Dai-Mei et al 2017^[43]^	Patients	Heart failure	Pathological fatigue	80 (38/42)	21.25%	Hospital	69.08 ± 13.48/71.44 ± 13.65	Baduanjin	Usual care	12 wk, 3, 35 min	PFS
Li et al 2020^[44]^	Patients	Prolonged cognitive activity	Physiological fatigue	66 (50/16)	0%/0%	Home	19.85 ± 2.39/19.70 ± 1.43	Baduanjin	Usual lifestyle	9 wk, 7, 30 min	FS-14
Liu et al 2020^[45]^	Patients	Neuromyelitis Optica spectrum disorders	Pathological fatigue	58 (55/3)	0%/0%	Hospital	40.38 ± 14.00/40.24 ± 15.06	Baduanjin	Usual care	12 wk, 5, 40 min	POMS
Zhao et al 2014^[47]^	Patients	Mental problems	Physiological fatigue	200 (92/108)	0%/0%	Home	19.8 ± 1.2/19.5 ± 1.7	Baduanjin	Usual physical activity	12 wk 5, 90 min	FS-14
Zhou et al 2020^[36]^	Patients	Using phones or working with head down for a long time	Physiological fatigue	62 (32/30)	0%/0%	Home	20.52 ± 0.14/20.35 ± 0.14	Baduanjin	Usual lifestyle	6 wk, 5, 30 min	FSS
Xie et al 2019^[48]^	Patients	Ankylosing spondylitis	Pathological fatigue	46 (11/35)	23.3%/23.3%	Home	18–60 yrs	Baduanjin	Usual lifestyle	Phase 1: 4 wk, 2 Phase 2: 8 wk, 3	BASDAI - Fatigue
Xiao et al 2016^[49]^	Patients	Parkinson disease	Pathological fatigue	96 (29/67)	6.3%/8.3%	Home	68.17 ± 2.27/66.52 ± 2.13	Baduanjin	Daily walking for not <30 min	24 wk, 4, 45 min	PFS-16

BASDAI = bath ankylosing spondylitis disease activity index, BFI = brief fatigue inventory, ChFs = Chalder fatigue scale, EORTC QLQ-C30 = European Organization for Research and Treatment of Cancer Quality-of-Life Questionnaire Core 30, FS-14 = fatigue scale-14, FSS = fatigue severity scale, MFI-20 = multidimensional fatigue inventory-20, MFSI-SF = multidimensional fatigue symptom inventory-short form, MPFS = modified piper fatigue scale, PFS-16 = 16-item Parkinson fatigue scale, POMS = profile of mood states.

### 3.3. Risk of bias

**Figure 2.**
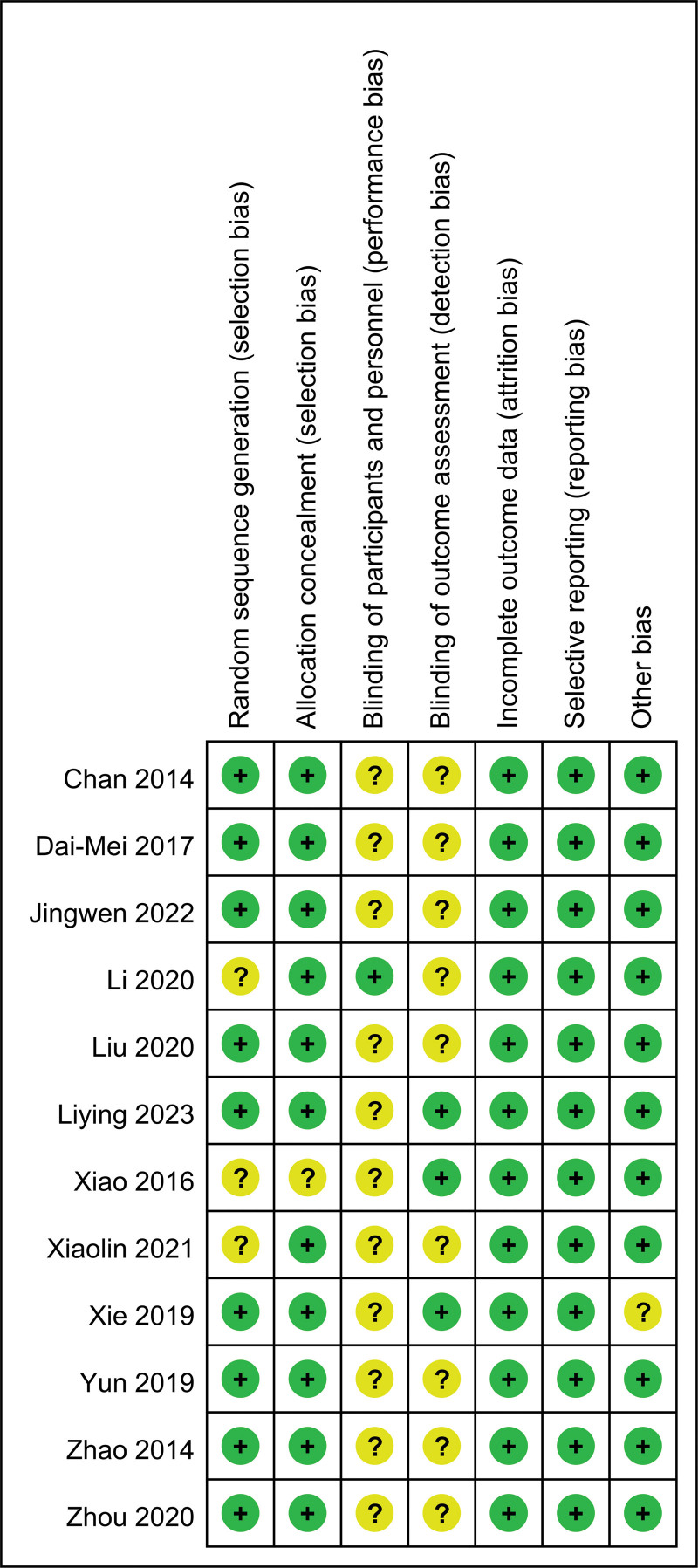
Risk of bias summary.

We evaluated the quality of each study using the Cochrane Collaboration risk-of-bias assessment tool. This tool assessed the risk of a study from 6 perspectives and marked it as having a high, low, or unclear risk (Fig. 2). Except for Liying study,^[35]^ none of the other studies reported blinding for performance bias during the intervention. It is difficult to determine whether, in these studies, knowledge of the assignment of the intervention to the participants affected the performance process and outcome assessment, and therefore, it can only be considered that there is an unclear risk of performance bias in these studies. Two studies did not describe the method of random assignment. Nine of the studies did not report whether the outcome data were assessed with blinding. All of these were considered unclear risks.

### 3.4. Synthesis of results

#### 3.4.1. Effects of Baduanjin on fatigue.

**Figure 3.**
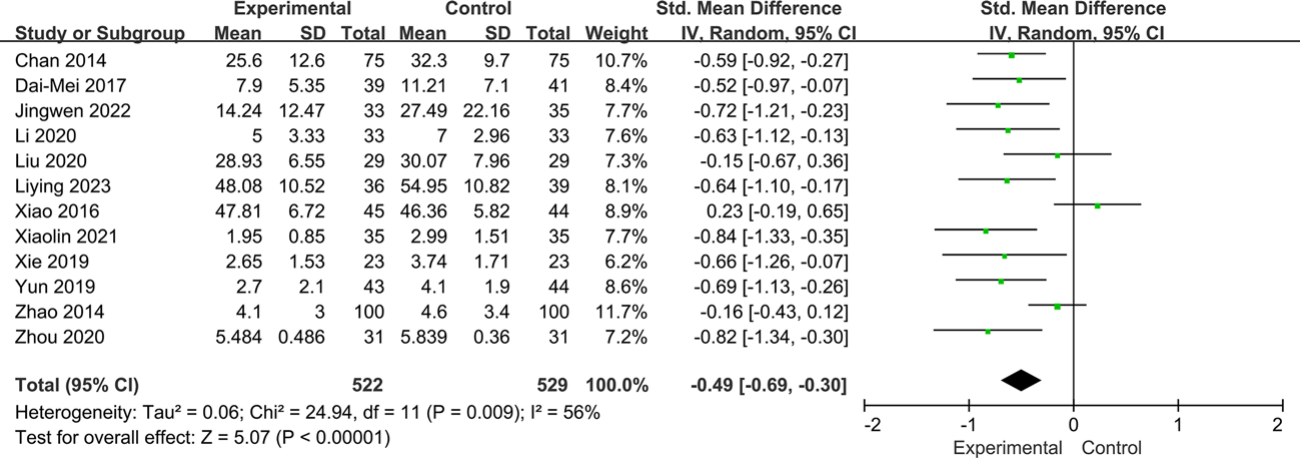
Forest plot of random effects model meta-analysis of the effect of Baduanjin on fatigue.

**Figure 4.**
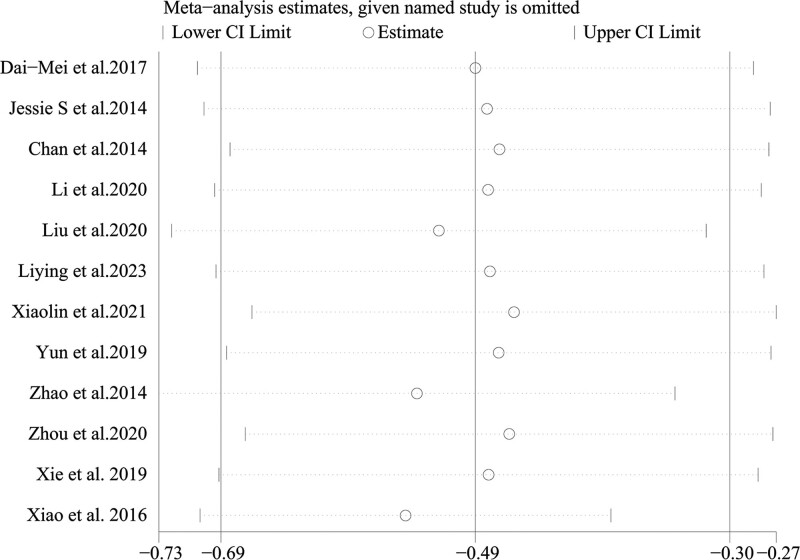
Sensitivity analysis of Baduanjin effect on fatigue.

Twelve RCTs including 1051 participants reported changes in fatigue intensity. In this cohort, 522 patients in the intervention group performed Baduanjin, and 529 patients in the control group received other treatments. Heterogeneity was insignificant (*I^2^* = 56%, *P =* .009; Fig. 3). Data analysis in the random effect method showed that Baduanjin could improve the symptoms of fatigue (SMD = −0.49, 95% CI = −0.69 to −0.30, *P =* .000; Fig. 3). A sensitivity analysis was conducted, and the result was robust (Fig. 4).

#### 3.4.2. Publication bias.

**Figure 5.**
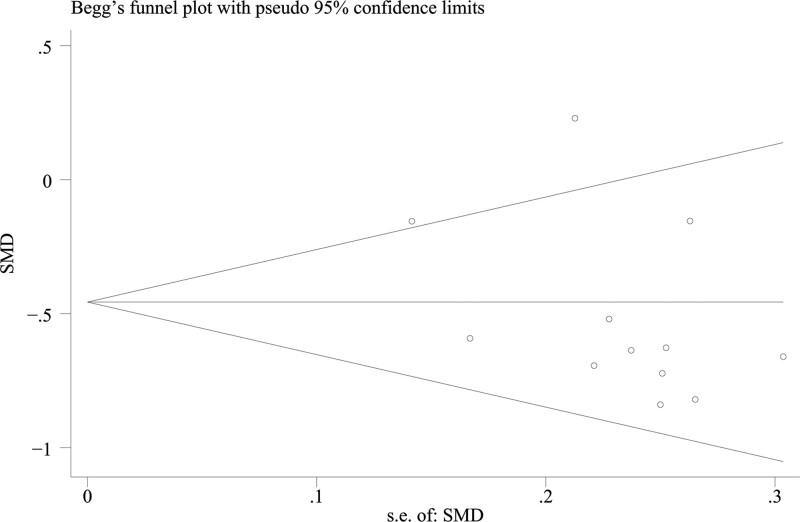
Funnel plot to evaluate the publication bias.

The funnel plot was symmetric. In Egger test, the publication bias was insignificant (*P* = .668). The results of these tests were not indicative of publication bias (Fig. 5).

#### 3.4.3. Meta-regression.

Factors examined individually in the meta-regression model of total fatigue intensity included duration, single intervention time, total time, number of interventions, the proportion of females, age, and practice location. No significant associations were found between fatigue intensity and the factors (Table 2).

**Table 2:** Meta-regression.

Variables	N	Coefficient	Standard error	*P*	95% CI
Duration	12	.0319367	.0139712	.062	−0.0022497–0.066123
Single intervention time	11	.0016337	.0059211	.791	−0.0123674–0.0156348
Total time	11	.0000954	.0000422	.828	−0.0000903–0.0001094
Number of interventions	12	−0.0030878	.0017733	.132	−0.0074268–0.0012512
Proportion of females	12	.0367953	.2992106	.906	−6953468–0.7689374
Age	11	.0037039	.0074437	.634	−0.0138976–0.0213055
Practice place	12	−0.4443211	.1432041	.021	−0.794729 to −0.0939132
Fatigue type	12	−0.2420908	.2134442	.300	−0.7643698–0.2801883

#### 3.4.4. Subgroup analysis.

**Figure 6.**
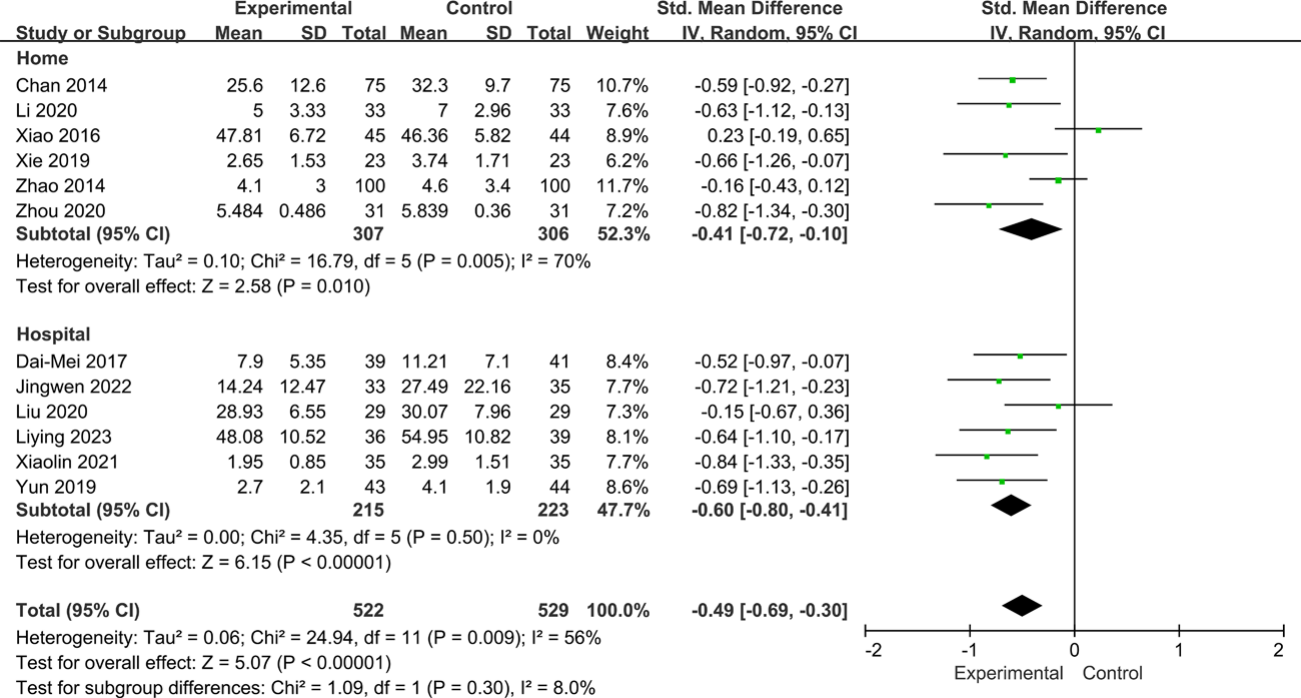
Subgroup analysis of the effect of practice location.

Although meta-regression did not detect significant factors responsible for heterogeneity, we selected practice location (such as home and hospital), age (<55 and ≥55 years), fatigue type (pathological fatigue and physiological fatigue), and duration (<12 and up to ≥12 weeks) for the subgroup analyses (Fig. 6).

In the subgroup analyses, we found that the overall effect sizes were relatively similar across the 4 groups.

For practice location, there were significant effects for training at home (SMD = −0.41, 95% CI = −0.72 to −0.10, *P* = .010; *I^2^* = 70%, *P* = .005; Fig. 6) and in hospital (SMD = −0.60, 95% CI = −0.80 to −0.41, *P* = .000; *I^2^* = 0%, *P* = .50; Fig. 6). However, there was no significant difference in the intervention effect of Baduanjin between training at home and in hospital (*P* = .30; Fig. 6).

**Figure 7.**
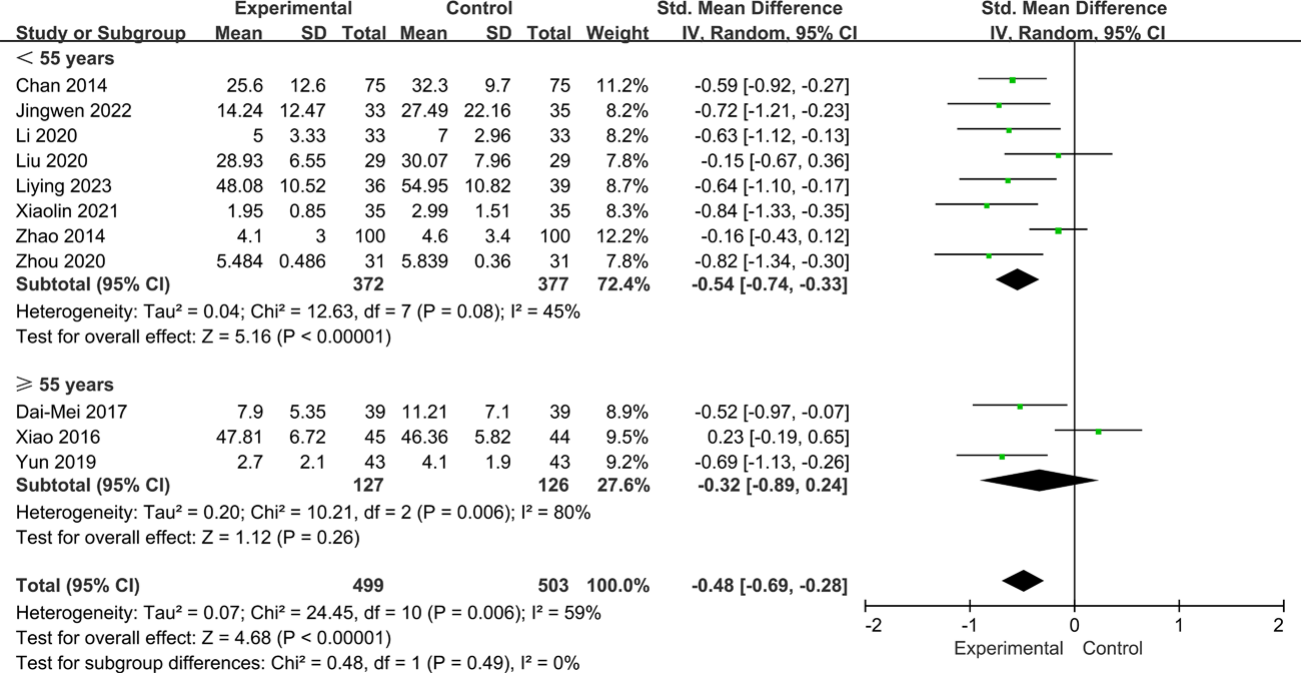
Subgroup analysis of the effect of different ages.

For different ages of participants, the Baduanjin had a significant effect on both groups of participants: mean ages <55 years (SMD = −0.54, 95% CI = −0.74 to −0.33, *P* = .000; *I^2^* = 45%, *P* = .08; Fig. 7) and ≥55 years (SMD = −0.32, 95% CI = −0.89–0.24, *P* = .006; *I^2^* = 80%, *P* = .006; Fig. 7). However, there was no significant difference in the size of intervention effect among participants of different ages (*P* = .49; Fig. 7).

**Figure 8.**
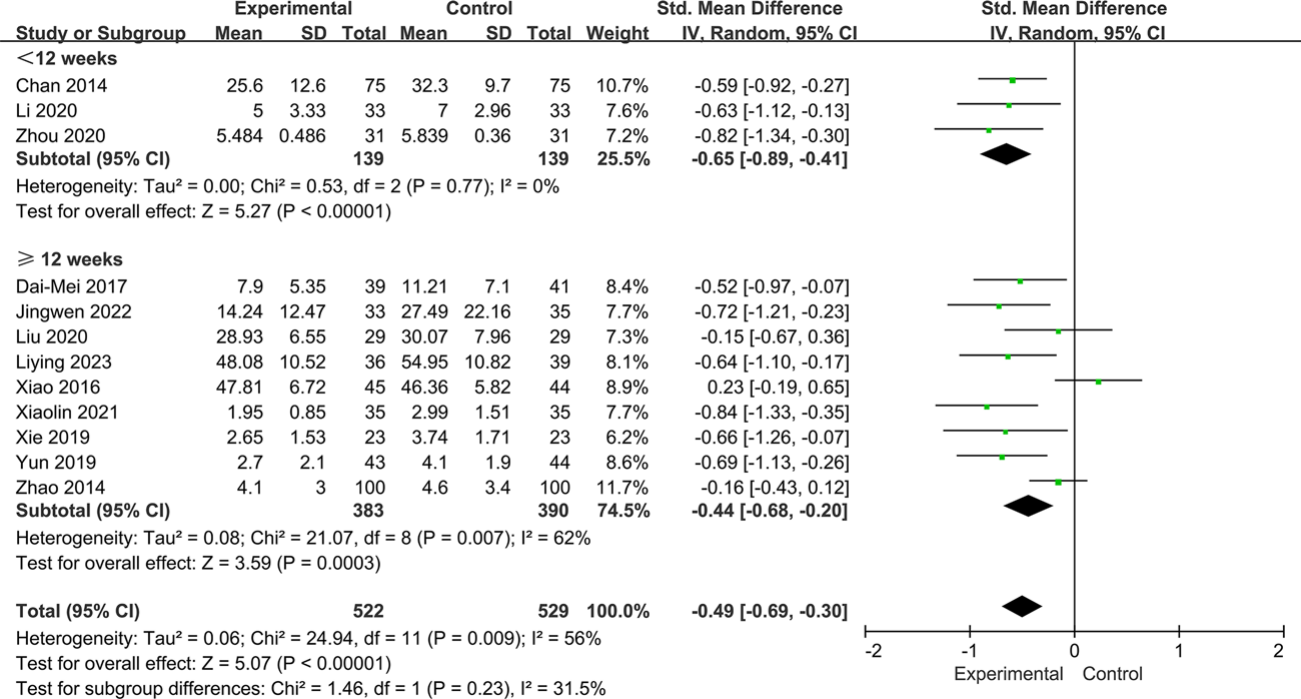
Subgroup analysis of the effect of different durations.

For intervention durations, significant effects were observed for both the <12 week (SMD = −0.65, 95% CI = −0.89 to −0.41, *P* = .000; *I^2^* = 0, *P* = .77; Fig. 8) and ≥12 week (SMD = −0.44, 95% CI = −0.68 to −0.20, *P* = .0003; *I^2^* = 62%, *P* = .007; Fig. 8) Baduanjin interventions. In addition, no significant difference was observed in the intervention effect of different intervention weeks (*P* = .23; Fig. 8). Baduanjin had a relatively stable intervention effect.

**Figure 9.**
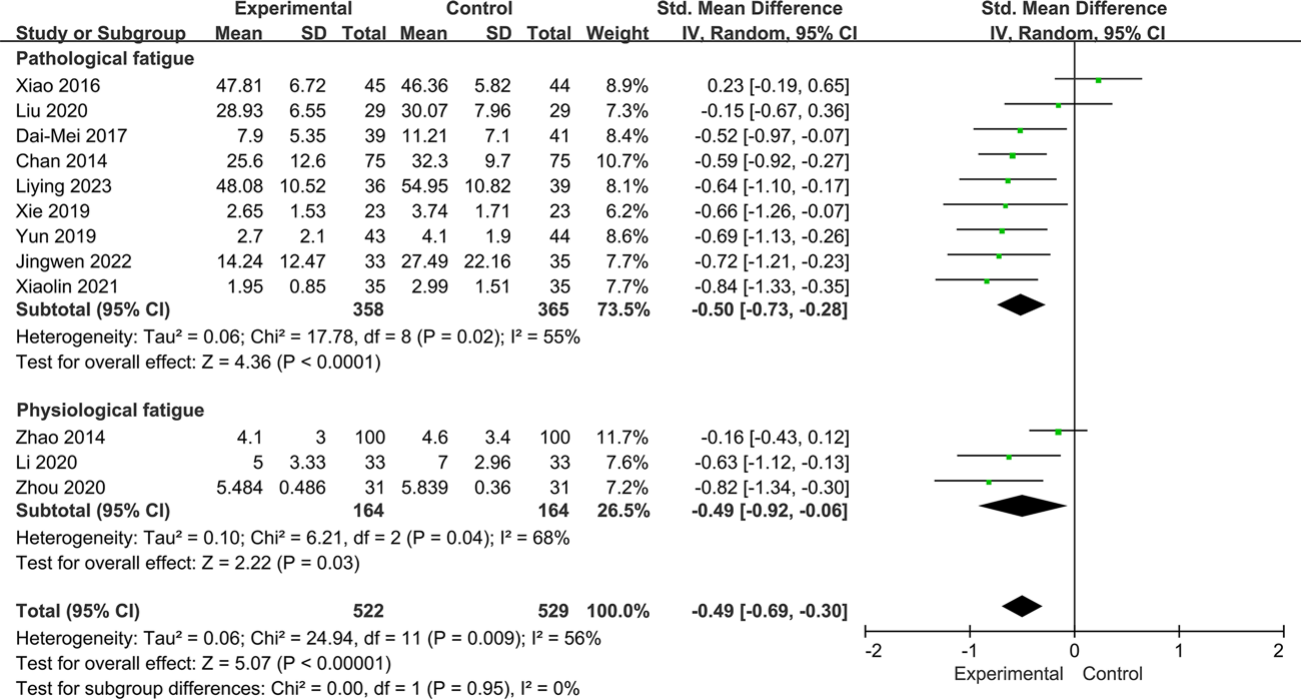
Subgroup analysis of the effect of fatigue types.

For fatigue types, there were significant effects for pathological fatigue (SMD = −0.50, 95% CI = −0.73 to −0.28, *P* = .000; *I^2^* = 55%, *P* = .02; Fig. 9) and physiological fatigue (SMD = −0.49, 95% CI = −0.92 to −0.06, *P* = .03; *I^2^* = 68%, *P* = .04; Fig. 9). However, there was no significant difference in the intervention effect of Baduanjin between training at home and in hospital (*P* = .95; Fig. 9).

Using the GRADE assessment tool (Table 3), we observed a moderate certainty of evidence for the effect of Baduanjin with participants 55-year younger, practicing in hospital, which was downgraded owing to some concerns with risk of bias. We observed low certainty of evidence for the effect of Baduanjin on fatigue, with different durations, fatigue types, participants 55-year or older, practicing in home, which was downgraded owing to some concerns with risk of bias and high heterogeneity.

**Table 3:** Summary of assessment of certainty of evidence (GRADE) for outcomes.

Outcome	Number of participants (studies)	Standardised mean difference (95%CI)	*I*^2^ (%)	Certainty of evidence (GRADE)
Fatigue	1051 (12)	−0.49 (−0.69, −0.30)	56	Low*^,^†
Subgroup analysis				
Duration <12 wk	278 (3)	−0.65 (−0.89, −0.41)	0	Low*^,^†
Duration ≥12 wk	773 (9)	−0.44 (−0.68, −0.20)	62	Low*^,^†
Participants <55 yr	749 (8)	−0.54 (−0.74, −0.33)	45	Moderate*
Participants ≥55 yr	253 (3)	−0.32 (−0.89, 0.24)	80	Low*^,^†
Home	613 (6)	−0.41 (−0.72, −0.10)	70	Low*^,^†
Hospital	438 (6)	−0.60 (−0.80, −0.41)	0	Moderate*
Pathological fatigue	723 (9)	−0.50 (−0.73–0.28)	55	Low*^,^†
Physiological fatigue	328 (3)	−0.49 (−0.92, −0.06)	68	Low*^,^†

CI = confidence intervals, GRADE = grading of recommendations assessment, development and evaluation, RCTs = randomized controlled trials.

* Some concerns with risk of bias.

† High heterogeneity.

## 4. Discussion

The results of this meta-analysis showed that Baduanjin has the potential to improve the overall intensity of fatigue in patients with a variety of diseases. In addition, the duration, frequency, number of interventions, sex, age, fatigue type, and practice location did not significantly affect the effectiveness of Baduanjin. Therefore, Baduanjin has the potential value of complementary therapy for the treatment of illness-related fatigue as the limitations are less.

To the best of our knowledge, this is the first systematic review and meta-analysis to specifically investigate the effects of traditional Chinese Baduanjin qigong on disease-related fatigue, providing significant evidence of the efficacy of qigong in the management of this condition. The quality assessment results of the included studies were deemed satisfactory, thereby establishing the basis for the reliability of the analysis results. The results of this study are similar to those of a systematic review and meta-analysis that suggested that traditional Chinese exercises, including qigong, could improve sleep quality.^[50]^ However, this systematic review and Meta-analysis conducted subgroup analysis of fatigue matching and found that Baduanjin had a certain improvement effect on fatigue, whether it is for pathological fatigue or physiological fatigue. However, it is noteworthy that only 1 study^[43]^ reported a completion rate of <80%. This might be attributed to factors such as the elderly population inadequate familiarity with distance learning devices, including mobile phones and computers; insufficient external assistance in receiving the intervention; or unexpected health deterioration.^[51–53]^ In addition, 1 study found that Baduanjin did not reduce the symptoms of fatigue,^[36]^ possibly because the intervention was only 6 weeks long, which was significantly short.^[42,54]^ Post-trial follow-up data were not available for all the included studies; hence, the long-term intervention effects of Baduanjin remained unclear and need to be further explored in subsequent studies. In general, the feasibility of Baduanjin appeared to be high.

All the studies included in this analysis were RCTs, a widely recognized method for establishing causality in intervention research. Missing data were obtained through direct contact with the authors of the original texts, which is a rigorous approach used to ensure data completeness. Notably, none of the studies reported any serious adverse events, indicating the safety of the intervention under investigation. Furthermore, our quantitative analysis revealed low heterogeneity between studies, indicating that the effect sizes were relatively consistent across the included trials. To further ensure the robustness of our findings, we excluded certain studies in which patients with serious neurological diseases might have had impaired motor function, potentially confounding the effect of the intervention. The scales used to measure the outcomes of the included studies are well-recognized and widely used in clinical practice, such as the Brief Fatigue Inventory and the Multidimensional Fatigue Inventory-20. Given the variability in these scales, we employed a random effects model to account for potential discrepancies and ensure reliable results. Finally, we noted that while the measurement scales used in the selected studies were validated, there was substantial variation in the specific instruments used. This raises the possibility that outcomes were either overestimated or underestimated during the data merging process. Therefore, the results of this analysis should be interpreted with caution.^[55]^ To enhance the comparability and precision of outcomes, utilizing uniform scales for collecting symptom data across various studies is crucial.

Baduanjin boosts energy by increasing oxygen intake, stimulating blood circulation, and enhancing vitality, thus reducing fatigue.^[56]^ This ancient Chinese exercise promotes muscle & joint health, improves strength and flexibility, and reduces soreness and stiffness.^[27]^ Additionally, its calming and meditative aspects reduce stress, crucial for mental and physical fatigue because focusing on stress reduction improves overall energy and wellness.^[57,58]^

## 5. Strengths and limitations

Our study strictly adhered to the Preferred Reporting Items for Systematic Reviews and Meta-Analyses statement for systematic reviews and meta-analyses, including the registration of our review methods. We only considered RCT designs due to their high level of reliability. In addition, we employed meta-regression analysis to investigate the impact of potential confounding variables on fatigue in older patients with cancer.

Nonetheless, this study has several limitations. First, there were significant variations in the types of diseases studied, which may have introduced heterogeneity in the analysis, thus affecting the validity, accuracy, and usefulness of the clinical recommendations. Second, most of the studies lacked blinding, which may have led to performance and detection biases. However, such biases were inherent because the intervention group for Baduanjin exercises likely knew the treatment they received. Third, all the literature included in this study was from China, and the sample size was limited. Therefore, further investigations are required to assess whether Baduanjin exercises have similar effects on other populations. Fourth, the scales used in the included studies were nonuniform, leading to potential bias in outcome measures between studies. Fifth, the majority of studies suffered from a lack of blinding of participants and assessors.

## 6. Implications for future studies

Further research is necessary to investigate the impact of Baduanjin on elderly patients with cancer outside of the Asian populations, utilizing RCTs. To establish the most effective Baduanjin regimen, interventions of varying frequency, quantity, and duration should be conducted and compared. To provide a more comprehensive understanding of the effects of Baduanjin interventions, not only postintervention outcomes but also intermediate and long-term outcomes should be evaluated. To ensure accurate reporting of results, it is essential to develop prespecified protocols that address how to evaluate outcomes and prevent deviations from expected interventions in cases where participant blinding is not feasible.

## 7. Conclusion

Baduanjin can reduce fatigue symptoms with comparatively flexible requirements. There is a paucity of studies on the same disease type, long-term intervention, and outside the Chinese population; therefore, more well-designed, high-quality, large sample-size studies are warranted in the future.

## Author contributions

**Data curation:** Haoyu Liu, Siling Liu.

**Formal analysis:** Haoyu Liu.

**Funding acquisition:** Haoyu Liu.

**Investigation:** Haoyu Liu, Siling Liu.

**Methodology:** Haoyu Liu, Siling Liu, Bingquan Luo.

**Project administration:** Lu Xiong.

**Resources:** Haoyu Liu.

**Software:** Haoyu Liu.

**Writing – original draft:** Haoyu Liu.

**Writing – review & editing:** Haoyu Liu, Siling Liu.

## Supplementary Material


