# Supplementary material for: Efficacy of Baduanjin for treatment of fatigue: A systematic review and meta-analysis of randomized controlled trials
Source: Medicine (Baltimore). 2023 Aug 11;102(32):e34707. doi: 10.1097/MD.0000000000034707 (PMC10419663; doi:10.1097/MD.0000000000034707)
Supplement: Supplementary file 1 [file medi-102-e34707-s001.pdf]

Search Strategies

Web of science:

#1: ((((TS=(baduanjin)) OR TS=(buduanjin)) OR TS=("eight-section brocades")) OR TS=("eight-section exercises")) OR TS=("eight-treasured exercises")

#2: TS=(fatigue)

#3: TS=(random*)

#4: #1 AND #2 AND #3

Embase:

#1: 'breathing exercise' OR buduanjin OR 'eight-section brocades' OR 'eight-section exercises' OR 'eight-treasured exercises'

#2: fatigue AND random*

#3: #2 AND #3

Medline:

S1: TX baduanjin OR TX buduanjin OR TX "eight-section brocades" OR TX "eight-section exercises" OR TX "eight-treasured exercises"

S2: TX fatigue AND TX random*

S3: S1 AND S2

Pubmed:

#1: ((((baduanjin) OR (buduanjin)) OR ("eight-section brocades")) OR ("eight-section exercises")) OR ("eight-treasured exercises")

"baduanjin"[All Fields] OR "eight-section brocades"[All Fields] OR ("eight section"[All Fields] AND ("exercise"[MeSH Terms] OR "exercise"[All Fields] OR "exercises"[All Fields] OR "exercise therapy"[MeSH Terms] OR ("exercise"[All Fields] AND "therapy"[All Fields]) OR "exercise therapy"[All Fields] OR "exercise s"[All Fields] OR "exercised"[All Fields] OR "exerciser"[All Fields] OR "exercisers"[All Fields] OR "exercising"[All Fields]))

OR ("eight treasured"[All Fields] AND ("exercise"[MeSH Terms] OR "exercise"[All Fields] OR "exercises"[All Fields] OR "exercise therapy"[MeSH Terms] OR ("exercise"[All Fields] AND "therapy"[All Fields]) OR "exercise therapy"[All Fields] OR "exercise s"[All Fields] OR "exercised"[All Fields] OR "exerciser"[All Fields] OR "exercisers"[All Fields] OR "exercising"[All Fields]))

#2: fatigue

"sleep"[MeSH Terms] OR "sleep"[All Fields] OR "sleeping"[All Fields] OR "sleeps"[All Fields] OR "sleep s"[All Fields] OR ("insomnia s"[All Fields] OR "sleep initiation and maintenance disorders"[MeSH Terms] OR ("sleep"[All Fields] AND "initiation"[All Fields] AND "maintenance"[All Fields] AND "disorders"[All Fields]) OR "sleep initiation and maintenance disorders"[All Fields] OR "insomnia"[All Fields] OR "insomnias"[All Fields])

#3: random*

"random*"[All Fields]

#4: #1 AND #2 AND #3

CBMdisc:

"八段锦"[全部字段:智能] AND "疲劳"[全部字段:智能] AND "随机"[全部字段:智能]

CNKI:

八段锦 and 疲劳 and 随机 (Topic search)

Wanfang:

八段锦 and 疲劳 and 随机 (Topic search)
